# A Multi-Domain Simulation Study of a Pulsatile-Flow Pump Device for Heart Failure With Preserved Ejection Fraction

**DOI:** 10.3389/fphys.2022.815787

**Published:** 2022-01-25

**Authors:** Caglar Ozturk, Luca Rosalia, Ellen T. Roche

**Affiliations:** ^1^Institute for Medical Engineering and Science, Massachusetts Institute of Technology, Cambridge, MA, United States; ^2^Health Sciences and Technology Program, Harvard – Massachusetts Institute of Technology, Cambridge, MA, United States; ^3^Department of Mechanical Engineering, Massachusetts Institute of Technology, Cambridge, MA, United States

**Keywords:** heart failure with preserved ejection fraction, heart failure, mechanical circulatory support, ventricular assist devices, left atrial decompression pump, lumped-parameter modeling, finite element modeling, living heart model

## Abstract

Mechanical circulatory support (MCS) devices are currently under development to improve the physiology and hemodynamics of patients with heart failure with preserved ejection fraction (HFpEF). Most of these devices, however, are designed to provide continuous-flow support. While it has been shown that pulsatile support may overcome some of the complications hindering the clinical translation of these devices for other heart failure phenotypes, the effects that it may have on the HFpEF physiology are still unknown. Here, we present a multi-domain simulation study of a pulsatile pump device with left atrial cannulation for HFpEF that aims to alleviate left atrial pressure, commonly elevated in HFpEF. We leverage lumped-parameter modeling to optimize the design of the pulsatile pump, computational fluid dynamic simulations to characterize hydraulic and hemolytic performance, and finite element modeling on the Living Heart Model to evaluate effects on arterial, left atrial, and left ventricular hemodynamics and biomechanics. The findings reported in this study suggest that pulsatile-flow support can successfully reduce pressures and associated wall stresses in the left heart, while yielding more physiologic arterial hemodynamics compared to continuous-flow support. This work therefore supports further development and evaluation of pulsatile support MCS devices for HFpEF.

## Introduction

Heart failure with preserved ejection fraction (HFpEF) is a complex and multi-factorial condition of diverse etiology, arising from the inability of the heart to relax and fill adequately (Borlaug and Paulus, 2011; Borlaug, 2020). In HFpEF, symptoms of heart failure ensue despite a normal left ventricular ejection fraction (LVEF), defined as the fraction of blood ejected into the systemic circulation over the maximum left ventricular (LV) filling volume during one heart cycle. Although guidelines for the classification of heart failure are continuously evolving, newly revised nomenclature defines HFpEF as heart failure with LVEF ≥ 55% and LVEF ≥ 60% in men and women, respectively (Borlaug, 2016; Lam and Solomon, 2021). The pathophysiological derangements associated with HFpEF arise from profound structural changes of the heart, primarily due to loss of ventricular compliance (Weber et al., 1993; Borbély et al., 2005), and often exacerbated by autonomic imbalance (Florea and Cohn, 2014) and other comorbidities (Mishra and Kass, 2021). Hemodynamically, these changes primarily result in elevations in the end-diastolic pressure-volume relationship (EDPVR), indicative of LV stiffening, and in the corresponding rise in end-diastolic pressure and drop in end-diastolic volume and cardiac output (CO; Borlaug et al., 2009). In addition, elevated LV end-diastolic pressures are transmitted retrogradely to the left atrium (LA) driving LA remodeling and symptoms of atrial fibrillation, and ultimately to the pulmonary circulation, resulting in congestion and exercise intolerance (Upadhya et al., 2015).

To date, medical therapies that ameliorate symptoms of HFpEF and quality of life in these patients are scarce, spurring studies of the use left ventricular assist devices (LVADs)—initially developed for patients with heart failure with reduced ejection fraction (HFrEF)—for other phenotypes. For example, studies report the implantation of LVADs in patients with end-stage restrictive or hypertrophic cardiomyopathies, often with similar cardiac anatomy and hemodynamics as those associated with HFpEF. In these patients populations, LVADs were shown to result in a variety of adverse events, including atrial collapse. These events arise from providing continuous-flow support to restrictive or hypertrophic cardiac anatomies, such as those associated with HFpEF (Topilsky et al., 2011; Wynne et al., 2011; Muthiah et al., 2013). To overcome these complications, a variety of other devices, including atrial shunts, LV expanders, electrical stimulators, and mechanical circulatory support (MCS) devices, are currently under development specifically for HFpEF (Rosalia et al., 2021b). While these technologies are at various stages of development, none have received FDA approval. Among technologies under development are a variety of MCS devices that aim to restore adequate CO and LA pressures (LAP; Miyagi et al., 2021c). These include the Synergy System (HeartWare International), the CoPulse device, and the left atrial assist device (LAAD). The Synergy system is a continuous-flow pump driving blood from the LA to the aorta (i.e., LA cannulation) or from the LV to the aorta (i.e., LV cannulation). Computational studies have demonstrated hemodynamic improvements associated with this system, including increased CO and reduced LAP (Burkhoff et al., 2015). The CoPulse device is a valveless pump designed for implantation at the apex of the heart enhancing LV filling volume and pumping synchronously with the native heartbeat. Although *in silico* and *ex vivo* studies are encouraging, concerns remain regarding the durability of the flexible membrane constituting the device for long-term support, and *in vivo* studies are yet to be conducted (Granegger et al., 2019; Escher et al., 2020). Finally, the LAAD is a continuous pump for implantation at the mitral valve designed to decompress the LA and is the only one of these devices undergoing animal investigation. In these studies, the LAAD was shown to effectively increase CO and mean aortic pressure (MAP) and decrease LAP (Fukamachi et al., 2020; Kado et al., 2020a,b; Miyagi et al., 2021a,b).

Although the use of MCS devices remains promising, a substantial body of the literature on mechanical support for HFrEF has emphasized that continuous-flow solutions may result in suction events as described above, and other complications, including increased bleeding, thrombosis, degeneration of aortic wall tissue, right ventricular (RV) failure, and ventricular arrhythmias (Patel et al., 2015; Healy et al., 2016). Particularly, acquired von Willebrand disease has been described as a universal condition secondary to continuous-flow LVADs developed by the totality of LVAD recipients (Meyer et al., 2014) attributed to high-shear stresses which trigger the unfolding and proteolysis of the von Willebrand factor—an adhesive glycoprotein with hemostatic function—ultimately resulting in gastrointestinal and, less frequently, cerebrovascular bleeding (Yoshioka et al., 2017; Shalabi et al., 2021). Thrombus formation, typically occurring at the inflow or outflow cannulas or at the pump, has been broadly observed in continuous-flow devices, such as the HeartMate II continuous-flow pump (Kirklin et al., 2014). Although pharmacotherapy has been shown to mitigate thrombotic events, pump exchange is often recommended once clinical signs of thrombus formation manifest (Mehra et al., 2014). Degeneration of aortic wall tissue and subsequent aortic regurgitation likely arise from abnormal aortic wall stress and aortic flow hemodynamics induced particularly by continuous-flow devices (Bouabdallaoui et al., 2018). Compensatory mechanisms leading to thinning of the media aortic layer and proliferation of atrophic smooth muscle cells ultimately cause aortic root dilation and regurgitation (Nishimura et al., 1998). On the other hand, a recent study has highlighted that endothelial function seems to be preserved under continuous-flow support, which was shown to have only negligible effects on vascular reactivity (Cortese et al., 2021). Other adverse effects associated with continuous-flow pumps include RV failure and ventricular arrhythmias, both arising from excessive ventricular unloading (Sack et al., 2018; Gopinathannair et al., 2019).

**Figure 1:**
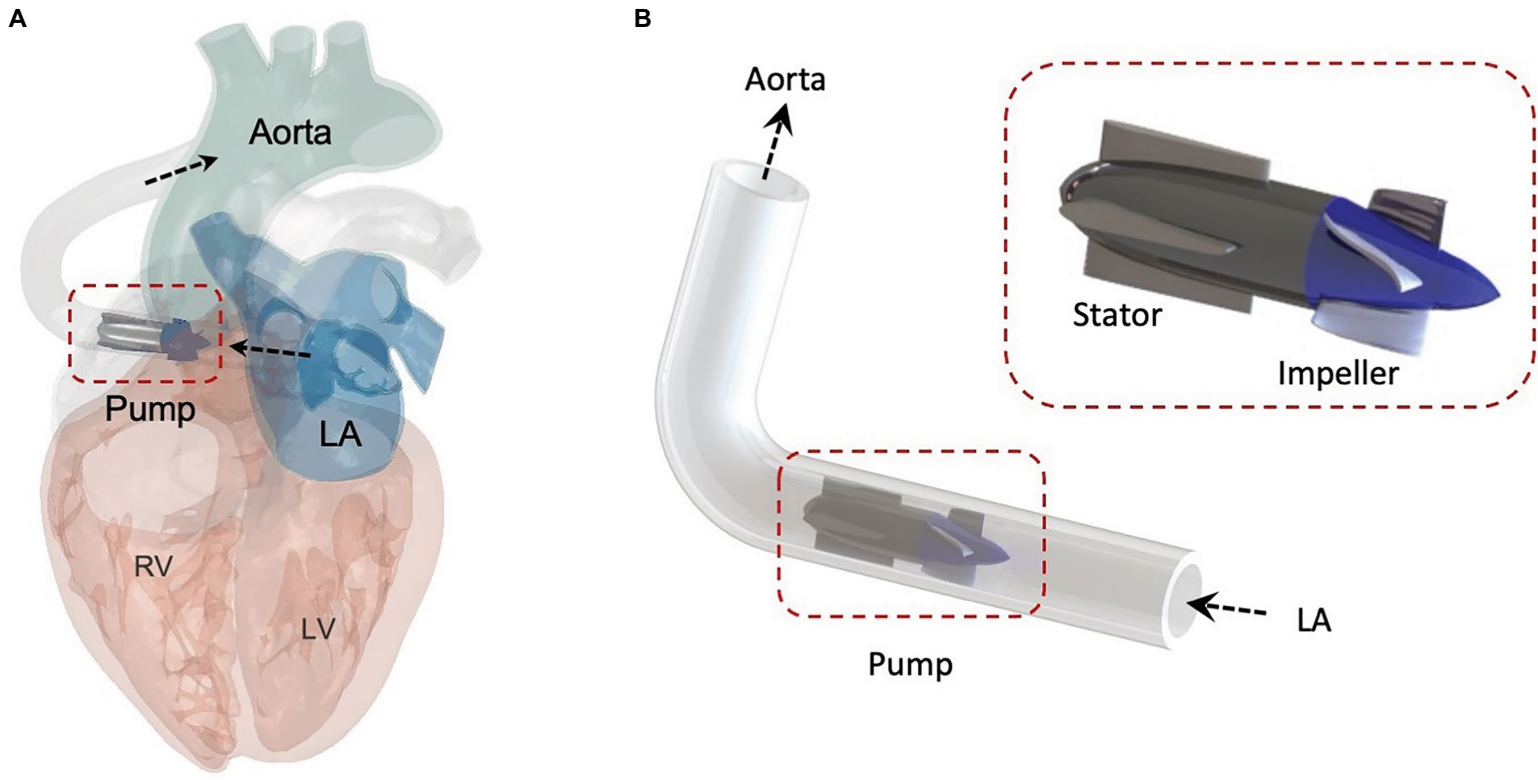
Schematic of the pulsatile pump for HFpEF. **(A)** Schematic of the pulsatile pump with LA cannulation, showing inflow from the left atrium (LA) and outflow into the aorta. **(B)** Illustration of the pump located between the LA and the aorta. LV, left ventricle; RV, right ventricle.

In this work, we investigate the feasibility of a pulsatile-flow pump with LA cannulation for HFpEF (Figure 1) and compare its performance with an analogous continuous-flow device, using a broad array of computational tools, including lumped-parameter (LP), computational fluid dynamics (CFD), and finite element analysis (FEA) platforms. By leveraging *in silico* tools to evaluate the efficacy of pulsatile-flow mechanical support for HFpEF patients and establish a framework for hemodynamic comparison with continuous-flow solutions, this research supports the development of MCS devices to enhance cardiac function and improve quality of life for patients with HFpEF.

## Materials and Methods

**Figure 2:**
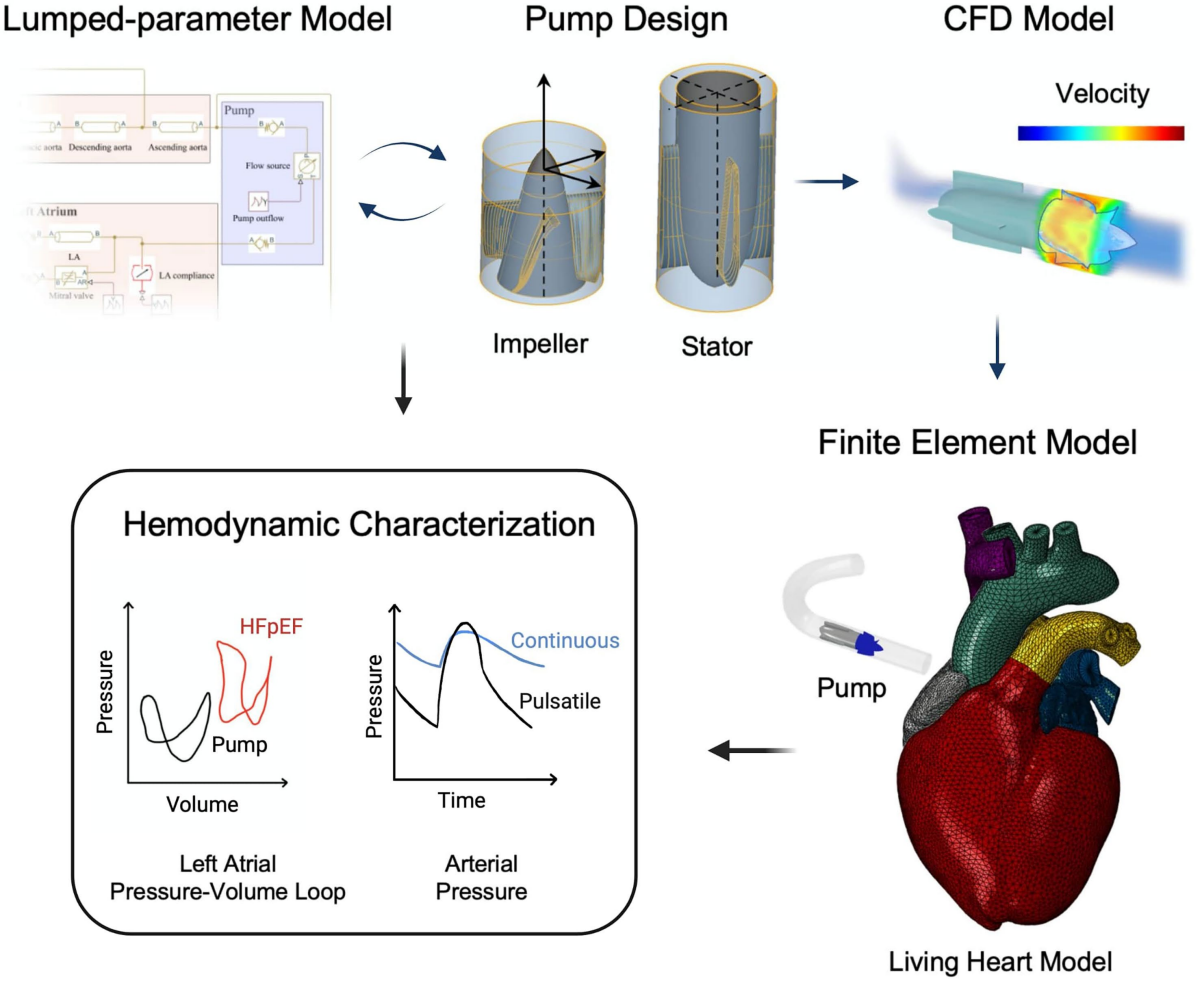
Workflow of pulsatile pump design and characterization. A lumped-parameter model is used for the optimization of the pump flow characteristics. CFD modeling enables evaluation of pump performance and hemocompatibility. Finally, integration of the pump into the finite element Living Heart Model allows comprehensive investigation of the resulting hemodynamics and comparison with findings from the lumped-parameter model.

Figure 2 illustrates the workflow of this study, which leverages a multi-domain computational framework for the development and characterization of a pulsatile MCS device for HFpEF with LA cannulation and in co-pulsation with aortic ejection. The hemodynamics resulting from support under other cannulation and pulsation modalities are shown in the Supplementary Material. Specifically, LP modeling enabled optimization of the pump outflow characteristics, CFD analysis allowed characterization of the hydraulic and hemolytic performance, and FEA modeling on the Living Heart Model (LHM, Simulia, Dassault Systèmes) was leveraged to characterize the hemodynamics and cardiac biomechanics resulting from both continuous and pulsatile-flow hemodynamic support in the HFpEF physiology. For continuous-flow support, the rotational speed of the pump was held constant, whereas a customized rotational speed profile was utilized for pulsatile-flow support. Findings from the LP and FEA models were compared for cross-validation. Target hemodynamics for the healthy and HFpEF physiologies and corresponding values obtained from the LP and FEA models are reported in the Supplementary Material.

### Lumped-Parameter Modeling

Cardiovascular hemodynamics were simulated on a lumped-parameter (LP) model developed on SIMSCAPE FLUIDS^™^ (MathWorks, R2020a). The physiologic and HFpEF phenotypes were recreated from work previously published by our group (Rosalia et al., 2021a,c). Specifically, HFpEF was modeled by reductions in the aortic valve orifice area and LV compliance to mimic pressure overload and LV stiffening, respectively (Rosalia et al., 2021a). An ideal flow source element that maintains a specified flow rate at its outlet was integrated between the LA and the aorta (LA cannulation), mimicking the behavior of the pulsatile pump (see Supplementary Material). The hemodynamics resulting from various flow rates were investigated to optimize pump output to provide adequate LA decompression. The amplitude and duration of sinusoidal-like outflow waveforms were varied to study the effects on LV, LA, and aortic hemodynamics, as summarized in the Supplementary Material.

Following optimization of the pump outflow, the hemodynamics resulting from pulsatile-flow support were compared with the healthy heart and HFpEF simulations. Further, additional simulations were performed by replacing the pulsatile pump with an analogous continuous pump with the same average flow output, enabling comparison between pulsatile- and continuous-flow mechanical support with LA cannulation for HFpEF.

### Pump Design

Figure 1 presents a simplified illustration of the pump, designed to draw blood from the LA into the aorta (i.e., LA cannulation), aiming to reduce elevated LAP and provide systemic hemodynamic support. The pump geometry is comprised of a rotating impeller with four blades and a stator with a flow straightener guided by four straight blades. The impeller was designed to be compact in size, with an overall length of 38 mm and a diameter of 12 mm. The 3D geometry of the pump was modeled using the CFturbo software based on standard turbomachinery design theory (CFturbo Software and Engineering GmbH, 2021). The axial impeller design frame was constructed taking the following parameters into consideration; the design point flow rate, pressure head, and the specific speed parameters. Based on the ideal outflow characteristics determined by LP modeling, the hydraulic profiles of the impeller and of the stator were generated and extracted from the CFturbo software.

### CFD Method and Performance Characterization

Computational fluid dynamics analysis was carried out on XFlow software (XFlow 2020, Dassault Systèmes), using a Large-Eddy Simulation turbulence model and the lattice Boltzmann formulation. Traditional numerical methods utilize finite volume and finite element procedures to solve the Navier–Stokes equations. Despite their wide usage, Navier–Stokes solvers suffer from drawbacks, such as complex meshing and highly empirical turbulence modeling. Unlike conventional CFD techniques, the lattice Boltzmann formulation utilized in the XFlow solver offers significant benefits to complex mesh problems and standard turbulence models, such as the Reynolds-averaged Navier–Stokes equations (Holman et al., 2012; Chávez-Modena et al., 2020). In this simulation, blood was modeled as a Newtonian incompressible fluid with a density of 1,050 kg/m^3^ and a viscosity of 0.0035 Pas. The pump was divided into three parts: the inlet, the impeller, and the stator. The rotating wall boundary condition was applied to the impeller region, while the stator was assigned as the stationary element. Constant pressure head values of 80, 100, 120, and 140 mmHg were used as boundary conditions between the inlet and outlet ports to measure the volumetric flow rate of the pump. The pump performance characteristic curves were generated using the flow rate as a dependent on the pressure gradient. Turbulence was simulated using the wall-adapting local eddy-viscosity model, as this offers consistent local eddy-viscosity and near-wall behavior. In this study, the hydraulic performance of the pump was characterized at various rotational speeds (22–30 k rpm) and for a set of constant pressure heads (80–140 mmHg), while a viscous scalar shear stress (SSS) was calculated for hemolytic characterization. The SSS parameter was computed based on the method proposed by Bludszuweit (1995). The shear stress field was therefore quantified using the shear rate formulation by calculating the derivatives of the velocity field (see Supplementary Material). Further, the particle tracking approach was leveraged to determine the residence time, which indicates the amount of time blood is located within the pump domain (Taskin et al., 2012). Three different grid sizes (i.e., the lattice resolution of 1.0 mm, 0.6 mm, and 0.4 mm) were used to test for grid independence. The stability of the numerical scheme, respect of the Courant–Friedrichs–Lewy condition, was monitored with the stability parameter for the convergence. The convergence of the stability parameter was achieved with a gap of 1% between the medium quality grid and the fine grid, maintaining the stability factor around 0.275. Combining convergence criteria and computation speed, the medium grid (i.e., 0.6 mm resolution and ~ 600,000 elements) was considered grid independent. The refinement method with 0.15 mm resolution was applied near the blade walls to ensure enough lattice elements as a boundary layer. Analysis was completed in ~18 h on a desktop PC with a 3.0 GHZ i7-9700 processor with 8 cores and 32 GB RAM.

### Finite Element LHM

The effects of the pulsatile pump were investigated on a dynamic cardiac FEA model conducted on Abaqus 2018 software (Simulia, Dassault Systèmes). The model, described in our previous work (Rosalia et al., 2021a), was readapted from the existing Living Heart Model (LHM; Baillargeon et al., 2014). Nonlinear explicit dynamic analyses were performed to simulate the dynamic heart. To represent blood flow in the LHM simulation, each heart compartment and the systemic circulation were modeled as distinct hydrostatic fluid cavities (see Supplementary Material). In addition, a surface-based fluid cavity was defined for the pump component and incorporated into the blood flow model. The passive response of the aorta and the ventricles was modeled using an anisotropic hyperelastic material formulation proposed by Holzapfel and Ogden for cardiac tissue (Baillargeon et al., 2014). A time-varying elastance model was implemented to describe the active cardiac tissue mechanics (Baillargeon et al., 2014). Within the FEA model, a fluid exchange interaction model was used to link the pump input and outflow to the LA and aorta, respectively. The pressure-flow rate data derived from the CFD simulations were used to generate the fluid exchange properties. The volume rate leakage module was utilized to specify the volumetric flow rate of the pump as a function of the pressure difference. The FE model of the heart contained 208,561 linear tetrahedral elements and 47,323 nodes. After the healthy and HFpEF physiologies were simulated dynamically, the pump cavity element was included in the flow model and adjusted accordingly to achieve different pump outflow waveforms. The flow output extracted from the CFD simulations was set as a fluid exchange property for the pump element. The steady-state response of cardiovascular system was examined after three cardiac cycles producing <5% variation in the cavity pressures. The LV stress values were recorded at the end of diastole and at the peak of systole. Data are expressed as mean ± standard deviation.

## Results

Findings from the characterization of the hydraulic and hemolytic performance of the pulsatile pump, as well as its effects on the left heart hemodynamics and biomechanics are described in the subsections below.

### Pulsatile Pump Hydraulic and Hemolytic Performance

**Figure 3:**
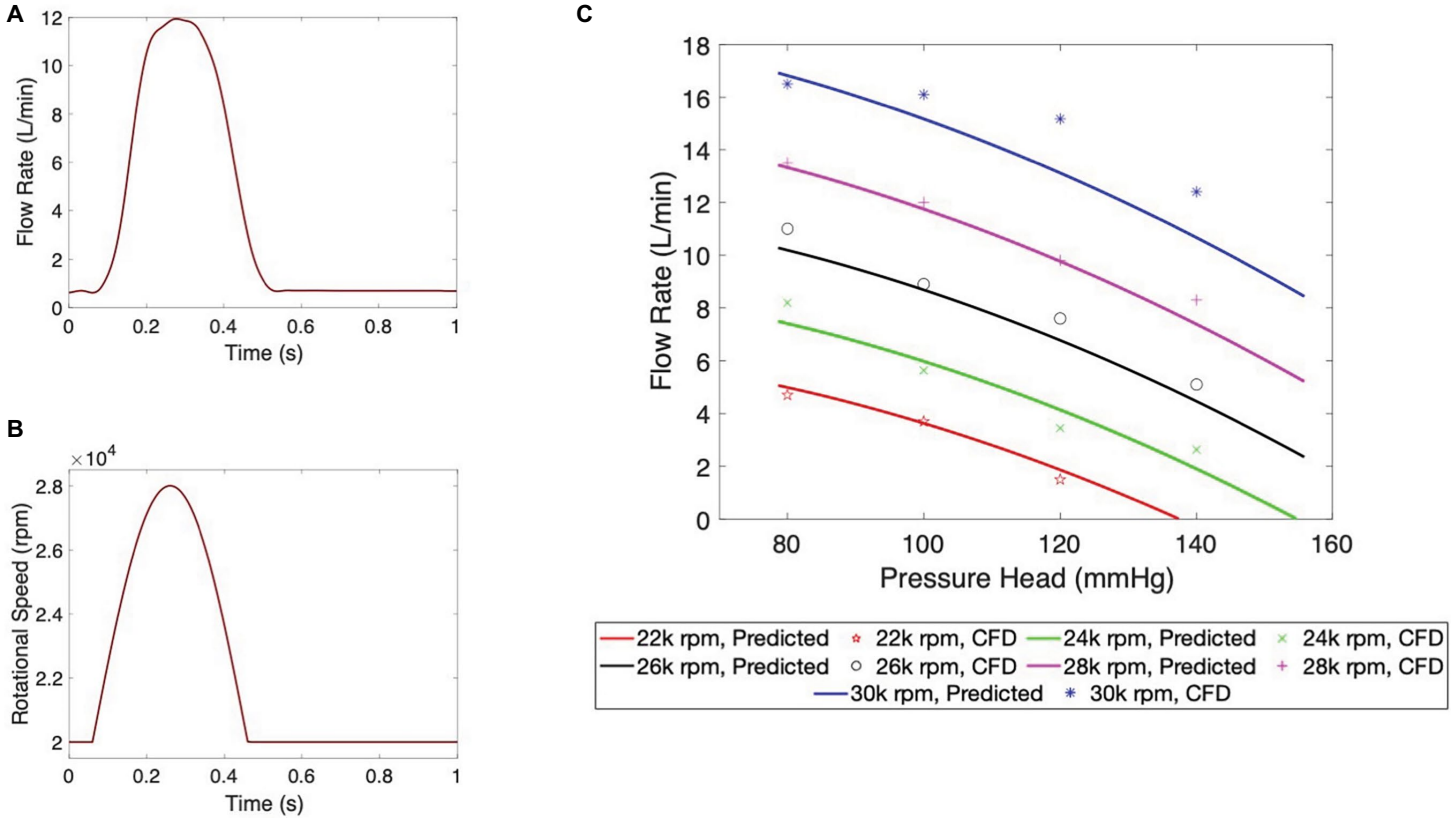
Pump hydraulic performance characterization. **(A)** Pump outflow and **(B)** impeller rotational speed waveform during an entire cardiac cycle. **(C)** Flow versus pressure head performance at five operating speeds. Continuous lines show analytical predictions obtained from the individual data points calculated by the CFD model.

The hydraulic and hemolytic performance of the pulsatile pump was evaluated by CFD modeling. Results of the hydraulic characterization are shown in Figure 3. Particularly, the flow rate at the pump output is depicted for the duration of one entire heart cycle in Figure 3A. From a baseline value of approximately 0.7 L/min during diastole, the flow rate reaches values of 11.9 L/min at peak ejection. A baseline rotational speed of 2,000 rpm was used to provide a minimum flow rate of 0.7 L/min at the beginning of the cycle to avoid any retrograde flow to the LA during diastole. The rotational speed of the impeller (Figure 3B) was determined by leveraging the pump pressure-flow rate curve and a sinusoidal-like outflow waveform to match the flow output. The pump outflow characteristics, including peak flow and ejection duration, were optimized using LP modeling. A subset of outflow profiles was further investigated using FEA to gain insights into both the hemodynamic and biomechanical effects of various levels of pulsatile support (see Supplementary Material). A sinusoidal waveform was used as this is known to yield superior hemocompatibility compared to other waveform morphologies (Nammakie et al., 2016). As shown in Figure 3B, pump ejection accounts for 40% of the cardiac cycle and is achieved by an increase in rotational speed from a baseline value of 20 k rpm during relaxation to 28 k rpm at peak ejection. Finally, the performance of the pump at five distinct peak rotational speeds is illustrated in the flow rate vs. pressure graph in Figure 3C. Notably, the designated operating conditions of 28 k rpm rotational speed at a pressure head of 100 mmHg correspond to the predicted flow rate value of 11.9 L/min.

**Figure 4:**
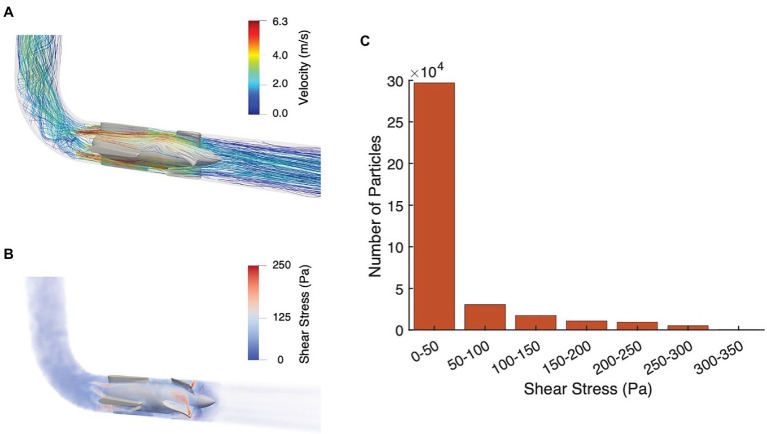
Pump hemolytic performance. **(A)** Flow streamlines and **(B)** Scalar shear stress (SSS) map with pulsatile-flow support during systole. **(C)** Distribution of blood particles subjected to a given shear stress.

The hemolytic characterization of the pulsatile pump is shown in Figure 4. Figure 4A illustrates the flow streamlines, while a map of the shear stress is depicted in Figure 4B. Results indicate a peak velocity of 6.3 m/s and a maximum SSS of 347 Pa in proximity of the stator and impeller blades, respectively. The flow streamlines were plotted using velocity contours instead of flow rate magnitude to illustrate the non-uniform distribution around the circumference of the impeller and provide a more detailed maps of the local flow characteristics. In addition, the shear stress distribution (Figure 4C) shows that only a relatively small fraction of blood particles (i.e., approximately 0.015%) is subjected to stresses greater than 250 Pa—known to result in hemolysis (Wood et al., 1999; Chen et al., 2019). Finally, the average residence time calculated from the pump inlet to outlet is 0.31 ± 0.4 s. Overall, these findings therefore suggest that, under the designated operating conditions, the pulsatile pump is associated with acceptable hemolytic performance.

### HFpEF Hemodynamics With Pulsatile-Flow Pump Support

**Figure 5:**
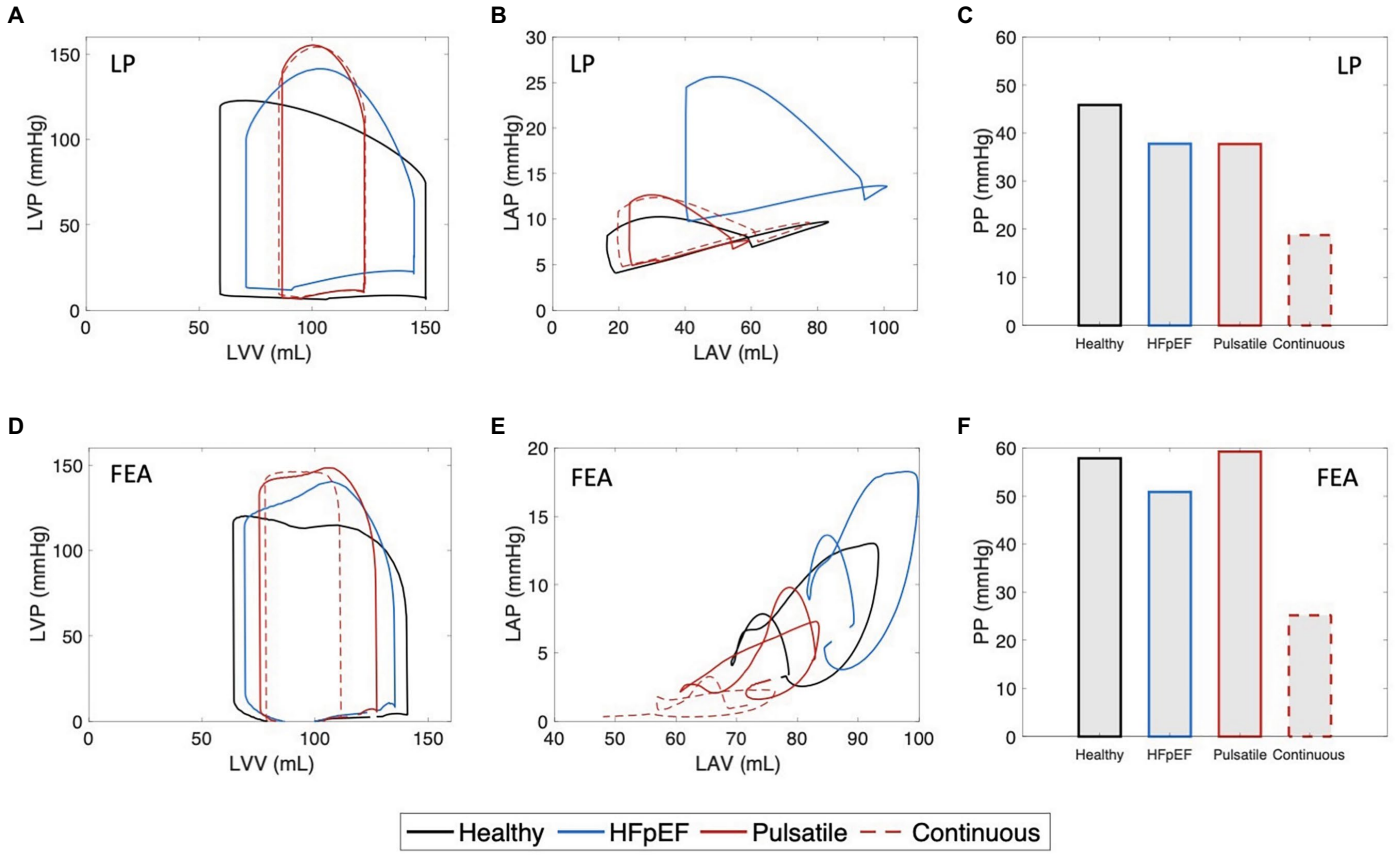
Hemodynamic results from lumped-parameter (LP) and finite element analysis (FEA) modeling. **(A)** Left ventricular (LV) pressure-volume (PV) loops, **(B)** left atrial (LA) PV loops, and **(C)** pulse pressure (PP) values obtained by LP model. **(D)** LV PV loops, **(E)** LA PV loops, and **(F)** PP values obtained by FEA model. Each plot shows comparison between the hemodynamics of the healthy heart, HFpEF, and under pulsatile and continuous pump support. LVP, left ventricular pressure; LVV, left ventricular volume; LAP, left atrial pressure; and LAV, left atrial volume.

The hemodynamic changes induced by pulsatile-flow support were evaluated by LP (Figures 5A–C) and FEA (Figures 5D,E) modeling. LV and LA PV loops and aortic pressures were obtained to enable comparison between the hemodynamics of the healthy heart, unsupported HFpEF, and HFpEF under pulsatile- and continuous-flow mechanical support, as shown in Figure 5 and summarized in Table 1. Results illustrate that both pulsatile- and continuous-flow support can successfully decompress the LA by significantly reducing both the peak and mean LAP (Figures 5B,E), yielding a corresponding drop in LV end-diastolic pressure (LVEDP), bringing values within the healthy physiological range (see Supplementary Material). These changes are known to ameliorate the adverse remodeling processes occurring in the LV and LA in HFpEF, which lead to symptoms of pulmonary congestion, exercise intolerance, and atrial fibrillation. However, while both types of support result in a further drop in SV and a raise in peak LVP from the HFpEF phenotype (Figures 5A,D), continuous pump support is also associated with diminished arterial pulsatility (Figures 5C,F). Conversely, pulsatile support results in maintained or improved pulsatility compared to the unsupported HFpEF phenotype.

**Table 1:** Comparison of hemodynamics at baseline, of the HFpEF phenotype, and of the HFpEF phenotypes with pulsatile or continuous support obtained by lumped-parameter (LP) and finite element analysis (FEA) modeling.

	Baseline		HFpEF		Pulsatile-flow support		Continuous-flow support	
	LP	FEA	LP	FEA	LP	FEA	LP	FEA
LAP_mean_ (mmHg)	8.00	5.63	14.66	12.71	7.58	7.88	8.65	3.43
PP (mmHg)	45.87	57.87	37.78	50.57	37.72	57.52	18.77	22.54
LVP_peak_ (mmHg)	122.87	120.33	141.64	134.13	155.33	139.84	154.35	144.96
LVEDP (mmHg)	7.30	4.57	22.49	16.20	10.60	12.33	10.34	5.41
SV (ml)	90.96	77.02	74.30	63.75	36.29	54.77	38.10	29.28

LAP_mean_, mean left atrial pressure; PP, pulse pressure; LVP_peak_, peak left ventricular pressure; LVEDP, left ventricular end-diastolic pressure; and SV, stroke volume.

### Cardiac Mechanics of HFpEF With Pulsatile-Flow Pump Support

**Figure 6:**
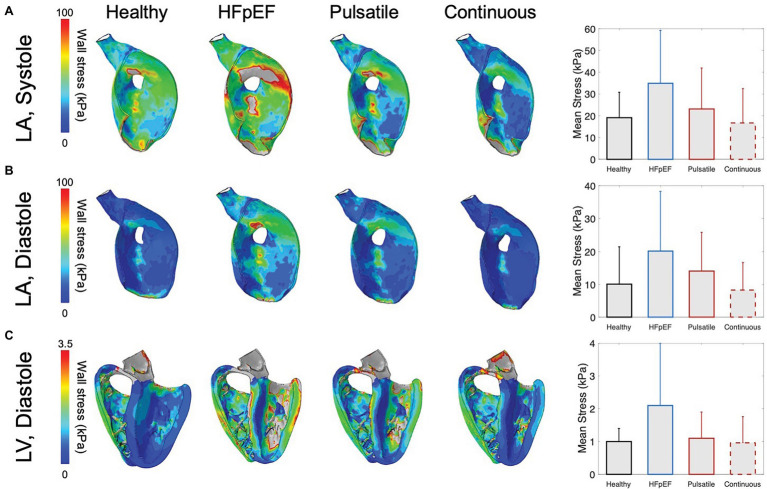
Wall stress obtained by finite element analysis (FEA). Stress distribution of the left atrial (LA) wall **(A)** during systole and **(B)** diastole, and **(C)** of the left ventricular (LV) wall during diastole in the healthy heart, HFpEF, and under pulsatile and continuous support. The gray color in the contour plots represents the over values of the range (maximum color bar value). Bar charts illustrate corresponding mean stress values of active LV elements (~123,000 elements) during systole and diastole. Error bars indicate +1 standard deviation from the mean.

Finite element analysis was further leveraged to evaluate the biomechanical response of the LV and LA in HFpEF under mechanical support (Figure 6). Consistent with the hemodynamic findings reported above, mechanical support yields a considerable reduction in LA wall stress during both systole (Figure 6A) and diastole (Figure 6B) and a corresponding drop in LV wall stress during diastole (Figure 6C), partly restoring the biomechanics of the healthy heart. Specifically, the mean LA wall stress drops from 34.9 ± 24.3 kPa (systole) and 20.2 ± 18.1 kPa (diastole) for unsupported HFpEF to 23.1 ± 18.9 kPa (systole) and 14.1 ± 11.7 kPa (diastole) with mechanical support, approximating mean stresses calculated for the healthy heart. Notably, under continuous support, the LV and LA likely experience suction or collapse events as indicated by further reductions in wall stress compared to those resulting from pulsatile support and by the remarkably altered LA geometry, particularly during the diastolic phase of the cardiac cycle. Negative principal stress was more prevalent in the LV septum wall and the LA wall regions during the continuous-flow support, which might be linked to potential suction or collapse occurrence (see Supplementary Material). While reducing the support provided could partially overcome this limitation, optimization of the outflow for continuous pumps is beyond the scope of the current work. During diastole, the mean LV wall stress is alleviated as it drops from 2.1 ± 1.9 kPa (HFpEF) to 1.1 ± 0.8 kPa with pulsatile support, approaching the value estimated for the healthy LV (1 ± 0.4 kPa). Changes in mean LV wall stress appear less significant during systole, as they primarily result from the active contraction of the myocardium, suggesting that these devices are unlikely to affect the native contractile function of the heart (see Supplementary Material).

## Discussion

In the context of MCS devices for heart failure, extensive research has been conducted to elucidate hemodynamic and pathophysiological differences arising from pulsatile- and continuous-flow support. To date, however, these studies have focused entirely on HFrEF, whereas investigations of the role that pulsatile-flow support may have on HFpEF patients have been broadly overlooked. This gap in the scientific literature has been emphasized by reports on the development of technologies that go beyond simple continuous-flow support, such as the PulseVAD (NorthernResearch, Oslo, Norway) device—a LA decompression pump with EKG-based adaptive outflow intended to adjust to patient-specific hemodynamics (Gude and Fiane, 2021). Despite the development of these systems for HFpEF patients, no study has yet described the hemodynamic and biomechanical effects that pulsatile support has on the HFpEF physiology.

In this work, we conducted a multi-domain computational investigation of the hemodynamic and biomechanical effects that pulsatile-flow support has on HFpEF and provided a comparison with continuous mechanical support. Specifically, we leveraged LP modeling to optimize the design of the pulsatile pump, evaluated its hydraulic and hemolytic performance by CFD analysis, and characterized arterial hemodynamics and both the hemodynamic and biomechanical response of the LA and LV using an FEA-based LHM simulation. All the computational approaches proposed in this work can be customized for patient-specific modeling in clinical applications. The choice between LP or FEA models largely depends on the availability of data on the hemodynamics and biomechanics, and on the desired model outputs. In general, LP models enable determination of a broader spectrum of hemodynamic measurements (including pulmonary and systemic circulation) at reduced computational costs. Examples of clinical metrics that can be used to validate the LP model include estimates of pressure and flow at the aorta, pulmonary artery, and vena cava. Additionally, as our LP model is defined in the hydraulic domain, measurements of any vessel diameter or anatomical cardiac valve orifice areas can be used as an input to our simulation. On the other hand, FEA models allow for higher resolution and more accurate evaluation of both the hemodynamics and biomechanics of the heart and local vasculature. Subsequently, metrics, such as LVEF, CO, LV pressures and volumes, and myocardial wall stiffness, can be leveraged for FEA model validation.

Our results demonstrate that both pulsatile and continuous support can provide adequate LA decompression, thus drive a reduction in LV diastolic pressures—commonly elevated in HFpEF. In this work, we demonstrate that, specifically for the HFpEF physiology, hemodynamic support with LA cannulation and in co-pulsation with aortic ejection yields better LA and arterial hemodynamics compared to LV cannulation or other pulsation modalities (see Supplementary Material). This work did not include an analysis of the coronary hemodynamics, and we acknowledge that counter-pulsation may provide benefits to coronary perfusion, as suggested by other studies investigating optimal pulsation modalities for the HFrEF physiology (Lim et al., 2009; Pirbodaghi et al., 2013). Our findings on the comparison of different cannulation options corroborate the literature on continuous-flow support for HFpEF in showing that LA cannulation yields better hemodynamics (Burkhoff et al., 2015).

This work shows that our proposed pulsatile pump may yield analogous changes in LAP (approximately 48%) to other devices currently under development for the treatment of HFpEF. For example, continuous-flow MCS solutions with LA cannulation have demonstrated a decrease in LAP ranging between 35 and 70% (Burkhoff et al., 2015), and other pulsatile VADs, such as the CoPulse pump, showed a decrease of 45–70% (Escher et al., 2020) depending on the degree of support. Both studies acknowledge the risk of adverse events including LA collapse at elevated levels of support. LV expanders are an alternate category of mechanical devices under development for the treatment of HFpEF and are associated with an LVED reduction of approximately 30% (Feld et al., 2006)—slightly lower than that resulting from our proposed approach (38.5%).

Further, the proposed pump demonstrated acceptable hemolytic performance and significant alleviation of wall stress in the LA during both systole and diastole, and a similar reduction of wall stress in the LV during systole suggesting it may attenuate the remodeling process associated with HFpEF. End-systolic and end-diastolic wall stress are generally estimated by measuring the LV wall thickness from cardiac magnetic resonance images. These estimates are based on Laplace’s Law, which assumes uniform chamber geometry and neglects the biomechanical properties of the myocardium (Wisneski et al., 2020). A population-based LV FEA study by Wang et al. (2016) highlighted that the end-diastolic wall stress at the mid ventricular region is greater in HFpEF patients (1.3 ± 0.2 kPa, *p* < 0.05) compared to the healthy control group (1.0 ± 0.2 kPa, *p* < 0.001). Consistent with this study, our findings showed wall stress values of 1.65 ± 0.8 kPa and 0.7 ± 0.1 kPa for the HFpEF and healthy physiology, respectively (Wang et al., 2016). As suggested by Alter et al. (2012), elevations in LV end-diastolic wall stress may induce LV hypertrophy and symptoms of HFpEF (Alter et al., 2012). These results illustrate similar trends in the LV wall stress between the healthy heart and the HFpEF physiology as well as its significance on disease progression. Investigations of cardiac biomechanics and alterations in LV wall stress may provide a better understanding of the HFpEF pathophysiology and of the efficacy of different MCS strategies.

The proposed computational framework could be utilized to evaluate the feasibility of other types of MCS devices. However, the modeling approaches developed in this work have some limitations. One of the major complications of continuous-flow MCS devices is the development of RV failure, induced by increased pulmonary vascular resistance and subsequent RV volume overload. The impact of the proposed pulsatile-flow MCS on the hemodynamics of the pulmonary vasculature and of the RV has not been investigated in this research. Analogously, the effects of CO changes resulting from LA unloading on the RV were not addressed and are beyond the scope of the current work. Future studies are warranted to optimize the pump design by performing parametric studies to further improve the hydraulic and hemolytic performance of the proposed design. A physical prototype of this device should be built to allow *in vitro* performance characterization and to enable *in vivo* investigation of the physiological interplay between LA unloading and RV physiology.

It is yet to be demonstrated whether pulsatile support in HFpEF effectively reduces the likelihood of complications associated with continuous support in HFrEF patients, such as organ bleeding, thrombosis, aortic wall tissue degeneration, and arrhythmias. Further investigation will reveal whether pulsatile support may be associated with additional advantages that are specifically relevant to the HFpEF physiology. Furthermore, the proposed models could not predict any possible regressions of the cardiac remodeling processes associated with HFpEF. Analogously, these platforms could not be leveraged to evaluate any chronic changes in the cardiac hemodynamics or biomechanics induced by pulsatile- or continuous-flow support. Growth and remodeling simulations or *in vivo* studies on a robust animal model of HFpEF will be necessary to evaluate the progression of the biological processes of HFpEF, as well as the safety and efficacy of pulsatile-flow support.

## Conclusion

This work models the hemodynamic effects of pulsatile-flow support with LA cannulation in the HFpEF physiology. A multi-domain computational framework, including LP, CFD, and FEA modeling, was developed to guide the design of the pulsatile pump and evaluate its hydraulic and hemolytic performance as well as the resulting LV, LA, and arterial hemodynamics. Results showed that the pulsatile pump here presented is poised to restore physiologic levels of LAP and diastolic LVP, as well as LA and LV wall stresses, demonstrating its potential to partially revert some of the hemodynamic and biomechanical derangements of HFpEF, and thus alleviate associated remodeling processes and symptomatology. In addition, comparison with an analogous continuous pump demonstrated that pulsatile-flow support yields more physiologic arterial hemodynamics in HFpEF, suggesting that pulsatile pumps may overcome some of the limitations currently affecting continuous-flow MCS solutions.

## Data Availability Statement

The raw data supporting the conclusions of this article will be made available by the authors, without undue reservation.

## Author Contributions

CO performed finite element and computational fluid dynamics simulations. LR performed the lumped-parameter analysis. All authors designed the study, and wrote and edited the manuscript.

## Funding

The authors acknowledge funding from the MathWorks Engineering Fellowship awarded to LR. CO is in part supported by a Fulbright-Turkey fellowship. ETR acknowledges support from the Department of Mechanical Engineering and the Institute for Medical Engineering and Science at the Massachusetts Institute of Technology.

## Conflict of Interest

The authors declare that the research was conducted in the absence of any commercial or financial relationships that could be construed as a potential conflict of interest.

## Publisher’s Note

All claims expressed in this article are solely those of the authors and do not necessarily represent those of their affiliated organizations, or those of the publisher, the editors and the reviewers. Any product that may be evaluated in this article, or claim that may be made by its manufacturer, is not guaranteed or endorsed by the publisher.
