# Supplementary material for: A Multi-Domain Simulation Study of a Pulsatile-Flow Pump Device for Heart Failure With Preserved Ejection Fraction
Source: Front Physiol. 2022 Jan 25;13:815787. doi: 10.3389/fphys.2022.815787 (PMC8822361; doi:10.3389/fphys.2022.815787)
Supplement: Supplementary file 1 [file Data_Sheet_1.docx]

**Supplementary Information**

Target hemodynamics of heart failure with preserved ejection fraction (HFpEF)

A detailed description of the lumped-parameter (LP) and finite element (FE) models used to simulate the hemodynamics of HFpEF is provided in a previously published work by our group^1^. In **Table S1**, we report some of the hemodynamic targets for the baseline and HFpEF physiologies. These target values were closely replicated by both our models^2–4^.

**Table S1|** Physiologic targets and corresponding values obtained by LP and FEA model for the healthy (baseline) and HFpEF phenotypes. CO: cardiac output; SV: stroke volume; LVP_peak_: peak left ventricular pressure; LVED: left ventricular end-diastolic pressure; LAP_mean_: mean left atrial pressure.

|  | Baseline | | | HFpEF | | |
| --- | --- | --- | --- | --- | --- | --- |
|  | Physiology | LP | FEA | Physiology | LP | FEA |
| CO (L/min) | 4.4 – 7.1 | 5.46 | 4.62 | 3.3 – 6.3 | 4.46 | 3.83 |
| SV (mL) | 51 – 111 | 90.96 | 77.02 | 40 – 95 | 74.30 | 63.75 |
| LVP_peak_ (mmHg) | 100 – 140 | 122.87 | 120.33 | >110 | 141.64 | 134.13 |
| LVEDP (mmHg) | 3 – 12 | 7.30 | 4.57 | >16 | 22.49 | 16.20 |
| LAP_mean_  (mmHg) | <12 | 8.00 | 5.63 | >12 | 14.66 | 12.71 |

Lumped-parameter model

*Domain*

A representation of the domain of the LP model is provided in **Figure S1**. Unidirectional valves were added across the flow source element to prevent retrograde flow.


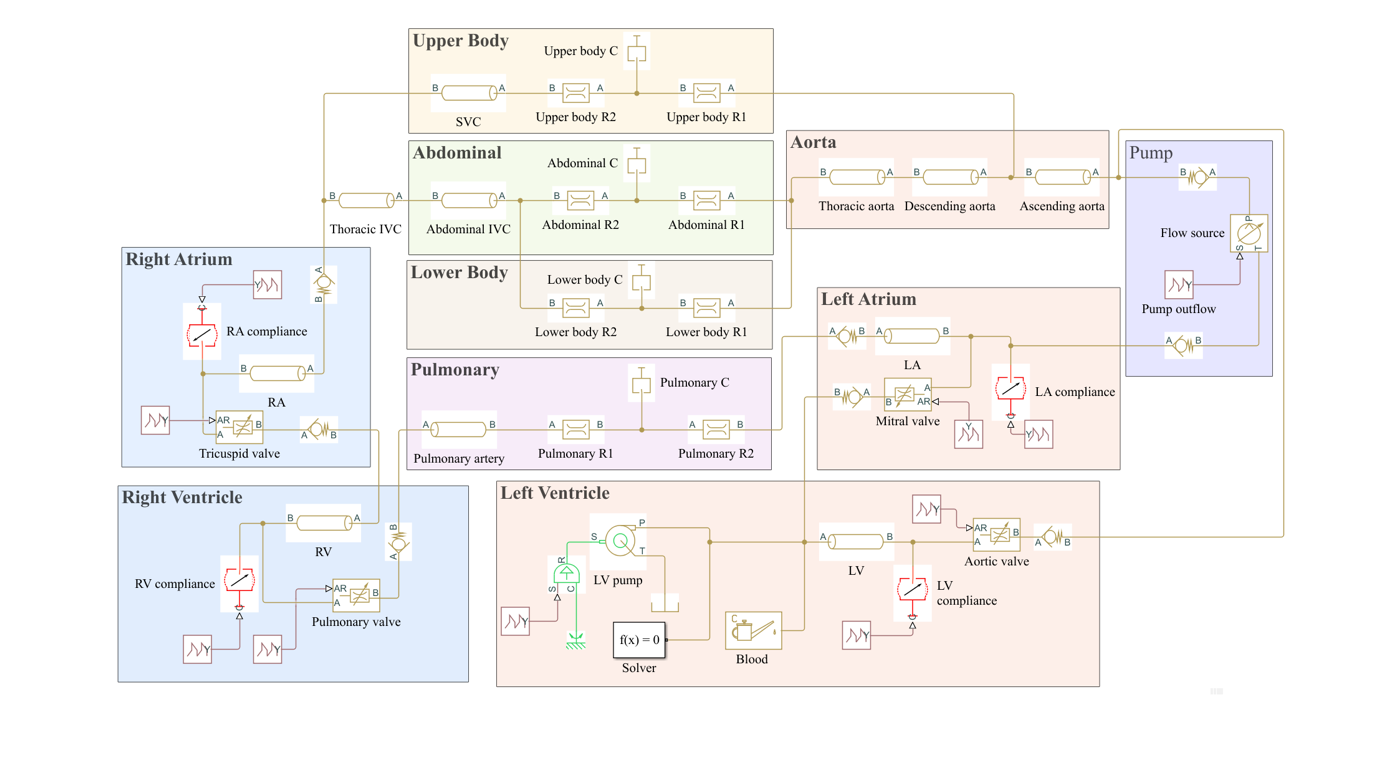


**Figure S1|** Schematic of lumped-parameter domain on SIMSCAPE FLUIDS^TM^.

*Pump outflow waveform analysis on lumped-parameter model*

A study of the hemodynamics resulting from varying the amplitude and ejection duration of the pump outflow sinusoidal-like waveforms was conducted to optimize support. Specifically, hemodynamic profiles were obtained at two distinct values of peak flow (q_max_: 7 and 14 L/min), while also varying the ejection duration (ED: 40%, 60%, 80%, 100% of one entire cardiac cycle). Representative curves are illustrated in **Figure S2A**. The peak of each of these waveforms was synchronized with peak systole for optimal hemodynamic performance.

**Figure S2B-E** shows the left ventricular pressure (LVP) and the aortic pressure (AoP) tracings, as well as the left atrial (LA) and left ventricular (LV) pressure-volume (PV) loops for these waveforms. As summarized in **Table S2**, these results show that higher peak flows and ejection durations yield lower mean LA pressures (LAP_mean_), indicative of greater LA decompression. However, elevated peak flows and ejection times also result in increased LV peak pressure (LVP_peak_) and AoP, and in a more significant drop in stroke volume (SV) and pulse pressure (PP), suggesting that intermediate support may be most suitable in ameliorating the hemodynamics of HFpEF.

**Figure S2|** Flow characteristics analysis on lumped-parameter model. **(A)** Representative pump outflow input tracings, with corresponding **(B)** left ventricular pressure tracings, **(C)** aortic pressure tracings, **(D)** left atrial pressure-volume loops, **(E)** left ventricular pressure-volume loops. q: flow; ED: ejection duration; LVP: left ventricular pressure; AoP: aortic pressure; LAP: left atrial pressure; LAV: left atrial volume; LVV: left ventricular volume. A, B, D, H in legends indicate the letters attributed to a given combination of peak flow and ejection duration (Table S2).

**Table S2|** Comparison of HFpEF hemodynamics with pulsatile support obtained on lumped-parameter (LP) model, for various pump peak outflows and ejection durations (A-J). LAP_mean_: mean left atrial pressure; PP: pulse pressure; LVP_peak_: peak left ventricular pressure; SV: stroke volume. A-D: 7L/min peak flow; E-H: 14 L/min peak flow. A,E: 40%; B,F: 60%; C,G: 80%, D,H: 100% ejection duration.

|  | LAP_mean_ (mmHg) | PP (mmHg) | LVP_peak_ (mmHg) | SV (mL) |
| --- | --- | --- | --- | --- |
| HFpEF | 14.66 | 37.78 | 141.64 | 74.30 |
| A | 11.35 | 39.19 | 146.81 | 59.37 |
| B | 10.63 | 34.55 | 149.03 | 52.67 |
| C | 9.57 | 29.12 | 151.42 | 45.99 |
| D | 8.54 | 23.58 | 153.91 | 39.32 |
| E | 9.02 | 41.62 | 152.11 | 45.07 |
| F | 6.66 | 31.96 | 156.84 | 32.36 |
| G | 4.44 | 27.16 | 162.45 | 20.16 |
| H | 1.27 | 18.62 | 170.12 | 9.26 |

Analysis of various pulsation modalities

In this work, we investigated the impact that pulsatile-flow support of a pump with LA cannulation in co-pulsation with the heart has on the hemodynamics of HFpEF. The LP model was leveraged further to study the effect of other modalities of pulsatile support, namely counter-pulsation with LA cannulation and co-pulsation with LV cannulation. Specifically, LVADs with counter-pulsation have been associated with some benefits in coronary perfusion for the HFrEF physiology^5,6^ and LV and LA cannulation approaches have been investigated in the context of continuous-flow support for HFpEF^2^.

**Figure S3A-C** provides a comparison of LV, LA, and aortic hemodynamics resulting from these other modalities of pulsatile support using our LP model. Our findings demonstrate that counter-pulsation results in adequate LA decompression, but it significantly compromises arterial pulsatility, which is remarkably reduced. Contrarily, LV cannulation was shown to successfully preserve arterial pulsatility, without, however, sufficiently reducing LA pressure. Overall, co-pulsation support with LA cannulation was demonstrated to reduce LA pressures while preserving arterial pulsatility, suggesting that this pulsatile-support modality may be the most successful in ameliorating the hemodynamics of HFpEF.

**Figure S3|** Hemodynamic results from the LP model with various modalities of pulsatile support, namely co- and counter-pulsation with LA cannulation and co-pulsation with LV cannulation. **(A)** LV PV loops, **(B)** LA PV loops, and **(C)** PP.

CFD shear rate formulation

The shear stress is computed as $\tau=\mu\cdot\dot{\gamma}$ where the shear rate ($\dot{\gamma}$) is obtained through the derivatives of the velocity field:

$$\dot{\gamma}=\sqrt{\begin{aligned} \left[ \frac{\partial u}{\partial x}\left( \frac{\partial u}{\partial x}+\frac{\partial u}{\partial x} \right)+\frac{\partial u}{\partial y}\left( \frac{\partial u}{\partial y}+\frac{\partial v}{\partial x} \right)+\frac{\partial u}{\partial z}\left( \frac{\partial u}{\partial z}+\frac{\partial w}{\partial x} \right) \right]+ \\ \left[ \frac{\partial v}{\partial x}\left( \frac{\partial v}{\partial x}+\frac{\partial u}{\partial y} \right)+\frac{\partial v}{\partial y}\left( \frac{\partial v}{\partial y}+\frac{\partial v}{\partial y} \right)+\frac{\partial v}{\partial z}\left( \frac{\partial v}{\partial z}+\frac{\partial w}{\partial y} \right) \right]+ \\ \left[ \frac{\partial w}{\partial x}\left( \frac{\partial w}{\partial x}+\frac{\partial u}{\partial x} \right)+\frac{\partial w}{\partial y}\left( \frac{\partial w}{\partial y}+\frac{\partial v}{\partial x} \right)+\frac{\partial w}{\partial z}\left( \frac{\partial w}{\partial z}+\frac{\partial w}{\partial x} \right) \right] \end{aligned}}$$

Finite element model

*Domain*

The lumped-parameter representation of the blood flow model in the LHM is presented in **Figure S4**. The pump chamber was added to represent the mechanical support with flow from the LA to the arterial chamber.


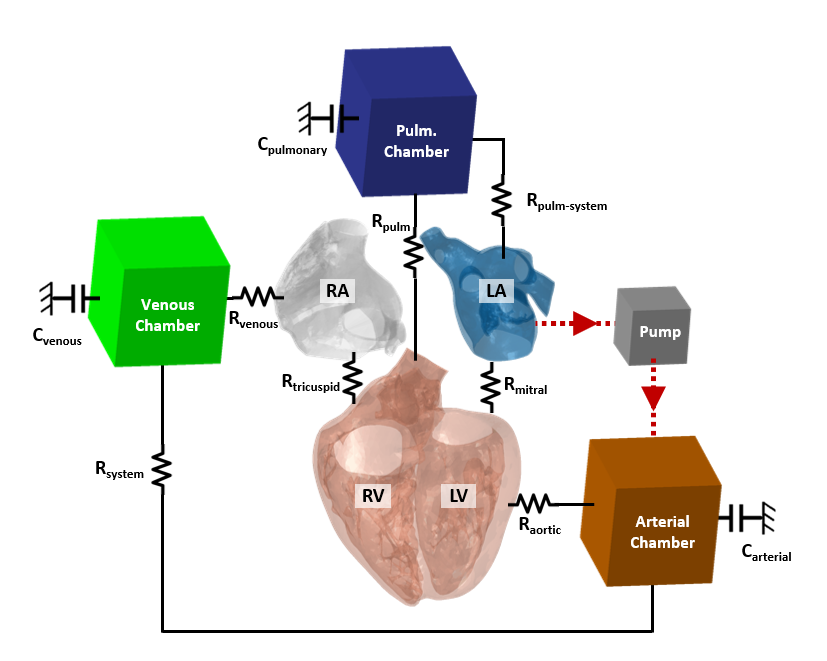


**Figure S4|** Schematic of lumped-parameter domain on the Living Heart Model (LHM).

*Pump outflow waveform analysis on finite element model*

The hemodynamic effects of various pump outflows, i.e., conditions A, B, D, and H (see above), were evaluated by finite element analysis (FEA). As FEA is more computationally intensive than lumped-parameter modeling, fewer cases were investigated. Conditions A, B, and D were selected to evaluate the effects of varying the ejection duration for a constant peak outflow, while condition H was chosen to study the role of peak outflow for a given ejection duration. Results, illustrated in **Figure S5** and summarized in **Table S3** are consistent with findings from our lumped-parameter simulation.

**Figure S5|** Flow characteristics hemodynamic analysis on finite element model. **(A)** Left ventricular pressure tracings, **(B)** aortic pressure tracings, **(C)** left atrial pressure-volume loops, **(D)** left ventricular pressure-volume loops. LVP: left ventricular pressure; AoP: aortic pressure; LAP: left atrial pressure; LAV: left atrial volume; LVV: left ventricular volume. A, B, and D, in legend indicate a peak flow equal to 7 L/min and ejection durations of 40%, 60%, and 100%. H in legend indicates a peak flow of 14 L/min and ejection duration of 100%.

**Table S3|** Comparison of HFpEF hemodynamics with pulsatile support obtained on finite element analysis (FEA), for various pump peak outflows and ejection durations (A, B, D, H). LAP_mean_: mean left atrial pressure; PP: pulse pressure; LVP_peak_: peak left ventricular pressure; SV: stroke volume. A, B, D: 7L/min peak flow; H: 14 L/min peak flow. A: 40%; B: 60%; D, H: 100% ejection duration.

|  | LAP_mean_ (mmHg) | PP (mmHg) | LVP_peak_ (mmHg) | SV (mL) |
| --- | --- | --- | --- | --- |
| HFpEF | 12.59 | 50.18 | 134.13 | 63.75 |
| A | 8.21 | 51.18 | 134.38 | 56.87 |
| B | 7.33 | 48.46 | 137.12 | 50.28 |
| D | 3.89 | 37.43 | 140.91 | 37.87 |
| H | 0.02 | 35.27 | 163.28 | 12.74 |

The effects of different pump outflow characteristics are reflected on the resulting cardiac biomechanics. **Figure S6** illustrates both the systolic and diastolic wall stress maps on the LA and LV for conditions A, B, D, H (see above). Increased levels of support result in remarkable changes in the geometry of the LA, exacerbating the risk of suction events.

**Figure S6|** Flow characteristics biomechanical analysis on finite element model. Stress distribution of the left atrial wall **(A)** during systole and **(B)** diastole, and of the left ventricular wall during **(C)** systole and **(D)** diastole for conditions A, B, D, and H (as per Table S3). Bar charts illustrate corresponding mean stress values. Error bars indicate + 1 standard deviation from the mean. A, B, D: 7L/min peak flow; H: 14 L/min peak flow. A: 40%; B: 60%; D, H: 100% ejection duration.

*Left ventricular systolic biomechanics due to pulsatile support.*

Evaluation of the left heart biomechanics demonstrated that pulsatile support could alleviate the elevated LA and LV wall stress commonly observed in HFpEF, as illustrated and discussed in the manuscript. For completeness, wall stress maps of the LV during systole resulting from optimal pulsatile support are shown in **Figure S7**. These plots illustrate that, in HFpEF, both pulsatile and continuous mechanical support have a negligible effect on the LV wall stress during systole, suggesting that these devices are unlikely to alter the native contractile function of the heart. Negative principal stresses of LV and LA are also shown in **Figure S7**.


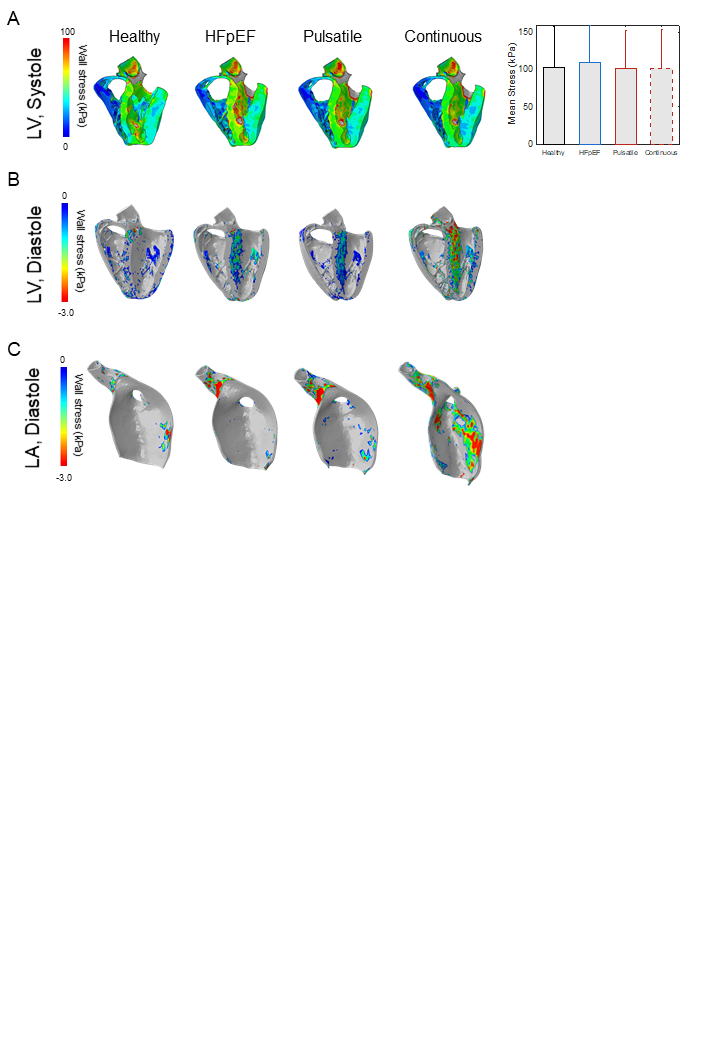


**Figure S7|** Wall stress obtained by finite element analysis (FEA). **(A)** Systolic left ventricular wall stress resulting from optimal pulsatile support. Bar chart illustrates corresponding mean stress values of active LV elements (~123,000 elements). Error bars indicate +1 standard deviation from the mean. **(B)** Negative principal stress distribution of the left ventricular (LV) and **(C)** left atrial (LA) wall during diastole.
